# Decreased dentin tubules density and reduced thickness of peritubular dentin in hyperbilirubinemia-related green teeth

**DOI:** 10.4317/jced.53490

**Published:** 2017-05-01

**Authors:** Rodrigo Neves-Silva, Fabio-Abreu Alves, Alberto Antunes, Mario-Fernando Goes, Marcelo Giannini, Maria-Dânia Tenório, José-Lécio Machado, Adriana-Franco Paes-Leme, Marcio-Ajudarte Lopes, Alan-Roger Santos-Silva

**Affiliations:** 1 Oral Diagnosis Department, Semiology Area, Piracicaba Dental School, University of Campinas (UNICAMP), Piracicaba, Sao Paulo, Brazil; 2 Department of Oral Medicine, A. C. Camargo Cancer Center, Sao Paulo, Brazil; 3 Restorative Dentistry Department, Dental Materials Area, Piracicaba Dental School, University of Campinas (UNICAMP), Piracicaba, Sao Paulo, Brazil; 4 Restorative Dentistry Department, Dentistry Area, Piracicaba Dental School, University of Campinas (UNICAMP), Piracicaba, Sao Paulo, Brazil; 5 Pediatric Dentistry Department, School of Dentistry, Federal University of Alagoas (UFAL), Maceio, Alagoas, Brazil; 6 Oral Medicine Department, School of Dentistry, Federal University of Alagoas (UFAL), Maceio, Alagoas, Brazil; 7 Mass Spectrometry Laboratory, Brazilian Biosciences National Laboratory – CNPEM, Campinas, Brazil

## Abstract

**Background:**

It is stated anecdotally that patients with liver diseases in childhood who develop green teeth have increased risk for rampant caries, which may be secondary to changes in dental structure. The aim of this study was to test the hypothesis that hyperbilirubinemia affects the dentin morphology of green teeth.

**Material and Methods:**

Sixteen primary teeth were prepared and divided into two groups (green teeth, n = 8 and control, n = 8), which were transversely fractured across the cervical third of the dental crowns; dentin was prepared and sputter-coated with gold, and examined under a scanning electron microscope. The mean density and mean diameter of dentin tubules, as well as the thickness of peritubular dentin, were compared.

**Results:**

Hyperbilirubinemia was associated with a decrease in the density of the dentin tubules (*p*< .01) and the thickness of peritubular dentin of green teeth (*p*< .01).

**Conclusions:**

There was a correlation between childhood hyperbilirubinemia and changes in the dentin morphology, including a decrease in the density of the dentin tubules and a reduction in the thickness of peritubular dentin in green teeth.

** Key words:**Hyperbilirubinemia, liver disease, childhood, dentin tubules, human teeth, scanning electron microscopy.

## Introduction

Metabolic diseases, local factors, and systemic factors may affect the color of teeth, causing permanent dental pigmentation (1). Several childhood diseases cause elevated serum levels of bilirubin (hyperbilirubinemia), which accumulate in tissue, such as bones, teeth, skin, and mucosa, and causing them to turn green. Other oral manifestations of hyperbilirubinemia include enamel hypoplasia, dental caries, and retardation of dental and bone development (2-4). The bilirubin accumulates in skin and mucosa only temporarily and is released due to rapid cell turnover; however, the formation of mineralized tissue permanently incorporates the bilirubin. Nonetheless, the pigmentation of the roots of developing teeth stops when the cause of the hyperbilirubinemia is treated, such as with a liver transplant (3,4).

Green teeth pigmentation caused by hyperbilirubinemia is mostly seen in the primary dentition (5-8). However, previous publications have reported green pigmentation in permanent dentition (9-11). Liver diseases, such as biliary atresia (BA) (3,12), hypoplasia of intrahepatic biliary tract (HIHBT) (13,14), and familial cholestasis (FC) (15) are the most commonly associated with green teeth. This study tested the hypothesis that hyperbilirubinemia is able to affect the dentin morphology of green teeth.

## Material and Methods

This research was approved by the local ethical committee for research (protocol number 122/2011).

-Clinicopathological study

**Figure 1:**
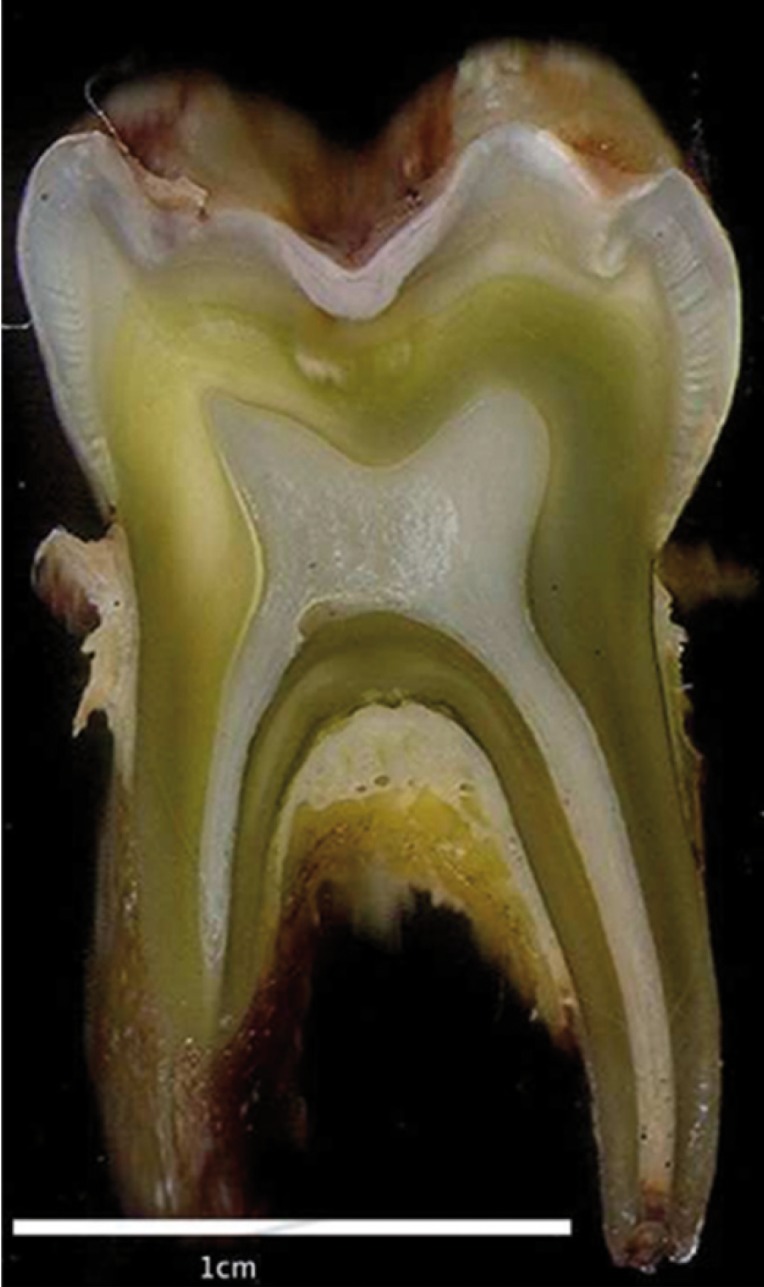
Macroscopic view of a longitudinal section of a green primary molar from one of the studied patients.

Demographic and medical data of patients enrolled in this study regarding gender, age, type of liver disease, liver transplant data and serum levels of bilirubin (direct, indirect, and total) were collected from the patients’ charts. Teeth analyzed in this study were obtained from patients who were indicated for tooth extraction prior to receiving a liver transplant at the Oral Medicine Depart-ment of AC Camargo Cancer Center, Sao Paulo, Brazil, and the teeth were stored in buffered solution (Fig. 1) (16). Teeth selected for this study did not present disseminated carious lesions or other macroscopic alterations that could compromise the analysis of dentin tissue.

-Mean density and mean diameter of dentin tubules and thickness of peritubular dentin

Sixteen primary green teeth were prepared and divided into two groups. In the group of green teeth, eight teeth were selected: two (25%) canines and six (75%) incisors. In the control group, eight teeth from healthy children were used: two (25%) canines and six (75%) incisors. The control teeth were matched with green teeth so that each group had the same amount of teeth in the same anatomical group and also paired by patients’ age. All teeth were prepared based on previous studies (17,18) and divided into subgroups. In Group 1, four green teeth were transversely fractured across the cervical third of the crown, and dentin was etched with 37% phosphoric acid (37 Condac, FGM Dental Products, Joinville, SC, Brazil) for 20 sec. In Group 2, four green teeth were transversely fractured across the cervical third of the crown, and dentin was not conditioned. In Group 3, four control teeth were transversely fractured across the cervical third of the crown, and dentin was etched with 37% phosphoric acid (37 Condac, FGM Dental Products, Joinville, SC, Brazil) for 20 sec. In Group 4, four control teeth were transversely fractured across the cervical third of the crown, and dentin was not conditioned.

The specimens were set in stubs with their fractured surface facing up by using colloidal silver adhesive (Electron Microscopy Sciences, Hatfield, PA, USA), sputter-coated with gold (Balzers SCD 050 sputter coater, Balzers Union Aktiengesellschaft, Furstentum Liechtenstein, FL, Germany), and examined in a scanning electron microscope (SEM; JSM–5600 LV - Jeol Ltd., Tokyo, Japan) operating at a voltage of 15 kV and using secondary electrons.

Fifteen SEM micrographs of fractured surfaces of each specimen were randomly obtained for the two groups at a magnification of 2,500x and analyzed in ImageJ 1.45s, following the methods of previously published articles (17,19).

To determine the tubular density, we counted all the dentinal tubules appearing in each micrograph. For each specimen, we used 15 electron micrographs measuring 175 mm x 114 mm. By using the scale bar appearing in each electron micrograph (35 mm = 10 µm), the dimensions of each micrograph corresponded to 50 and 32.57 µm, thus resulting in an area of 1628.5 µm2 (= 0.0016285 mm2). We divided the number of dentinal tubules present in each electron micrograph by that area (0.0016285 mm2).

To determine the diameter of dentinal tubules, 15 micrographs were obtained from each sample following the same pattern des-cribed above and basing our methods on those of Dutra-Correa *et al.*, (17). To take the measurements, we used a scale bar of each micrograph obtained from ImageJ 1.45s. Fifteen dentinal tubules were randomly selected in each micrograph and measured for their horizontal and vertical diameters, totaling 30 measurements per micrograph; therefore, a total of 450 measurements were performed for each specimen. The mean diameter of dentinal tubules of each specimen was then obtained from the average values of the 450 measurements. The mean values were compared among the subgroups (Group 1 versus Group 3 and Group 2 versus Group 4).

To avoid tubule density and diameter variation, we controlled the selection of the tubules by site sampling at the cervical third of the dental crowns and compiling identical dentin fracture levels in all groups. All samples were examined at the same angle of incidence at the SEM, aiming to avoid negative influences during the measurements of the diameter (circular versus ellipsoid diameter). In addition, sampling sites of dentin tubules in all groups were controlled by selection of the tubules to be analyzed.

We established the thickness of peritubular dentin by arriving at the difference between the mean diameter values of fractured-conditioned dentin tubules from the fractured dentin tubules (non-conditioned surface) for green teeth (Group 1 – Group 2) and for control teeth (Group 3 – Group 4). The differences in dentinal tubule diameters between the acid etched and the merely fractured specimens revealed an estimate of the thickness of the peritubular dentine “wall” removed in the former samples. All the results were statistically processed using EXCEL® software to obtain average values with their respective standard deviations.

-Statistical analysis

We performed a statistical analysis of the data concerning to investigate the mean density and mean diameter of dentin tubules and the thickness of peritubular dentin for the green and control specimens. A one-way ANOVA parametric nature test was applied using SAS® system with a significance level of 5% (α = .05).

## Results

-Clinicopathological study

The ages of the eight patients enrolled in this study ranged from 8 to 12 years with a mean age of 10 years; four were male and four were female. All patients had been diagnosed with hyperbilirubinemia in childhood; four had BA, one had primary biliary cirrhosis (PBC), one had HIHBT, one had FC, and one had liver cirrhosis (LC). Clinicopathological data are presented in  Table 1.

**Table 1:**
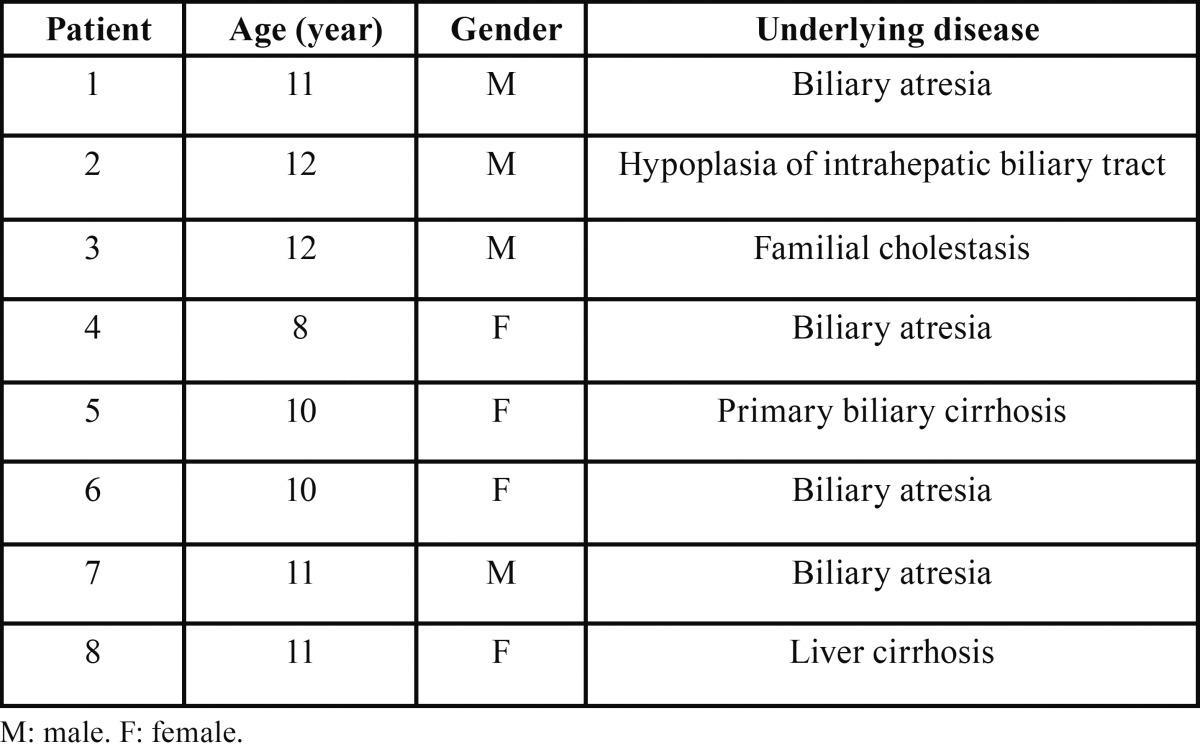
Clinical features of patients.

-Mean density and diameter of dentin tubules and thickness of peritubular dentin

**Figure 2:**
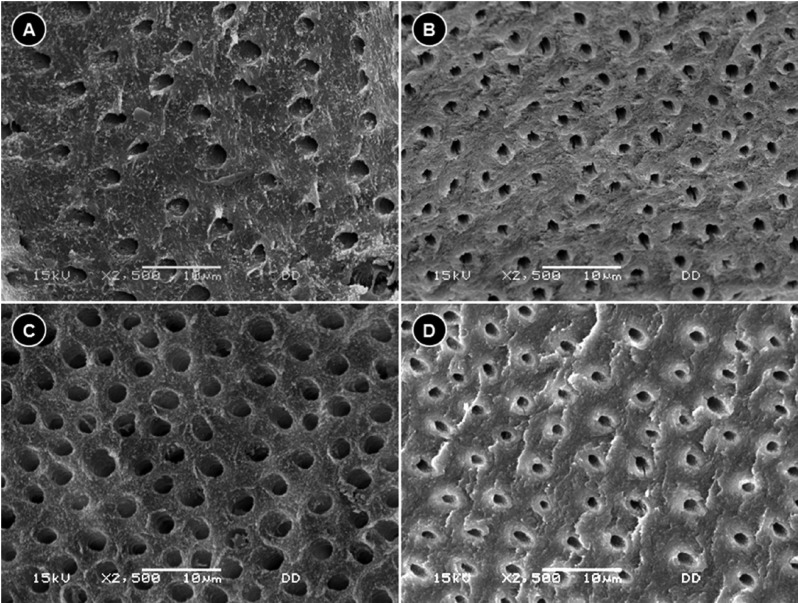
Density and diameter of the dentin tubules by SEM. A. Dentin tubules of Group 1. B. Dentin tubules of Group 2. C. Dentin tubules of Group 3. D. Dentin tubules of Group 4. SEM: scanning electron microscopy.

**Figure 3:**
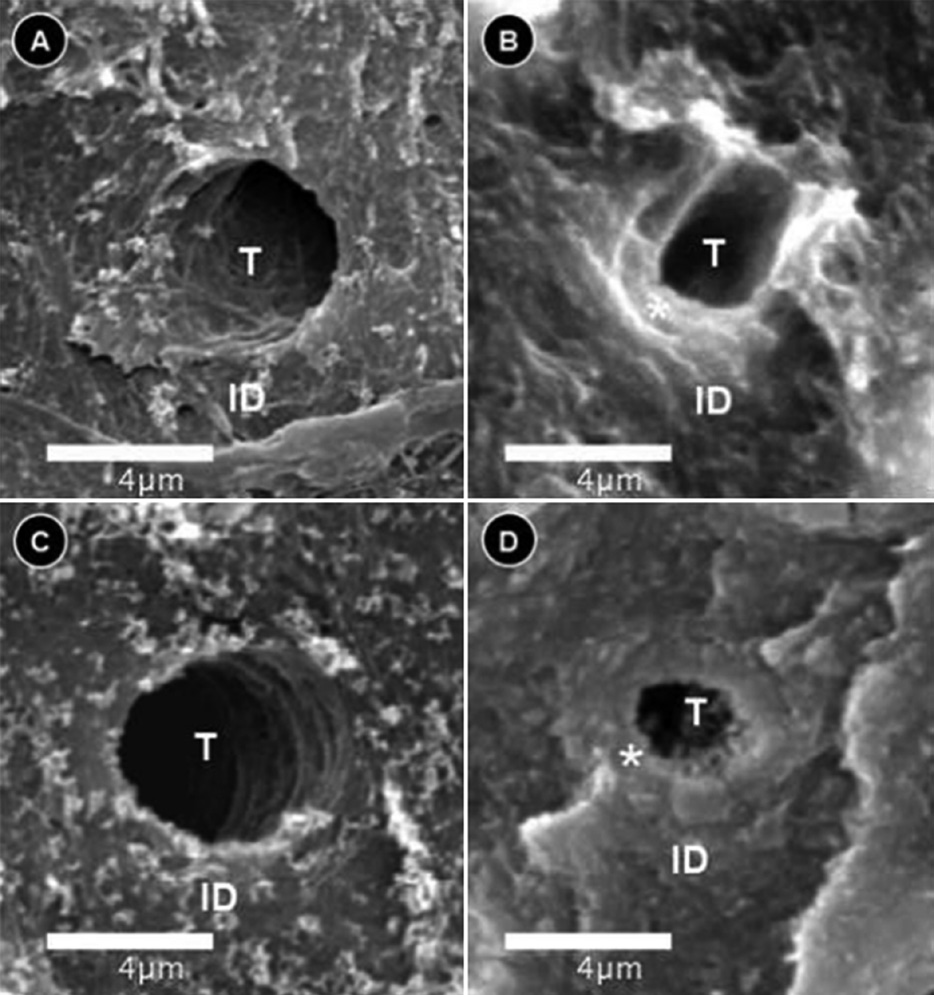
Peritubular dentin by SEM. A. Dentin tubule of Group 1. B. Dentin tubule of Group 2. C. Dentin tubule of Group 3. D. Dentin tubule of Group 4. ID: intertubular dentin, * = peritubular dentin, T: dentin tubule. SEM: scanning electron microscopy.

In transversely fractured green and normal teeth, the average values of mean density of the dentin tubules were obtained from measurements registered in each micrograph. The measures concerning the mean density of the dentin tubules varied from 15.1 to 20.6 (tubules/mm2) to groups I and II, respectively. The results revealed the following averages of the mean density of the dentin tubules to groups III and IV: 34.2 to 29.1 (tubules/mm2), respectively ( Table 2). The recorded average values of the mean diameter of the dentin tubules were 0.95 µm (group I), 0.78 µm (group II), 1.09 µm (group III), and 0.73 µm (group IV), ( Table 3). The authors also verified that there was a reduction in the thickness of peritubular dentin in green teeth group when compared to control group. The measures of the thickness of peritubular dentin varied from 0.13 to 0.24 µm in green teeth group, and varied from 0.25 to 0.42 µm in the control group ( Table 4). During the semi-quantitative analysis of peritubular dentin, there was no evidence of morphological changes or variations in its structural features (Figs. 2,3).

**Table 2:**
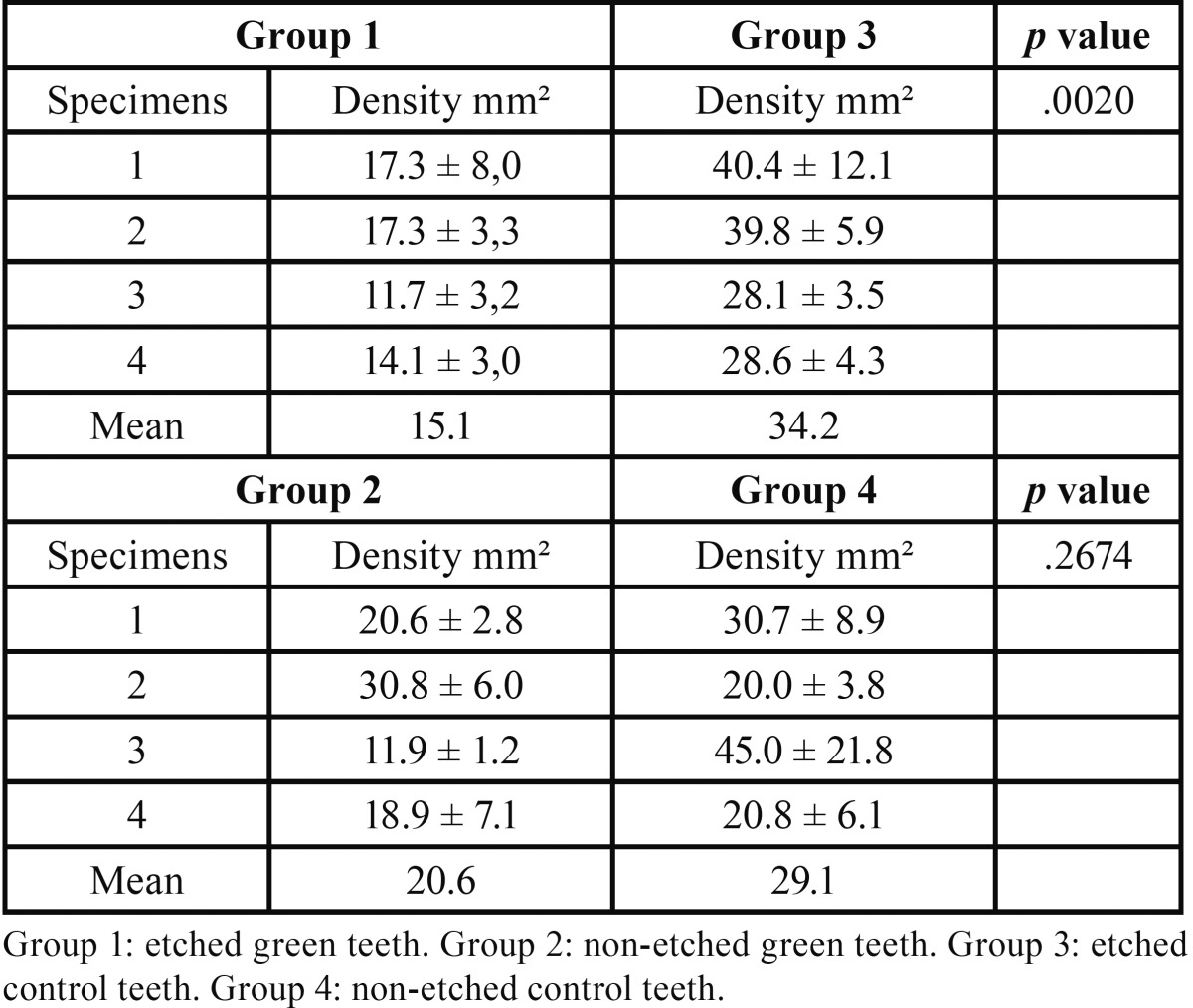
Mean density of the dentin tubules (tubules/mm2).

**Table 3:**
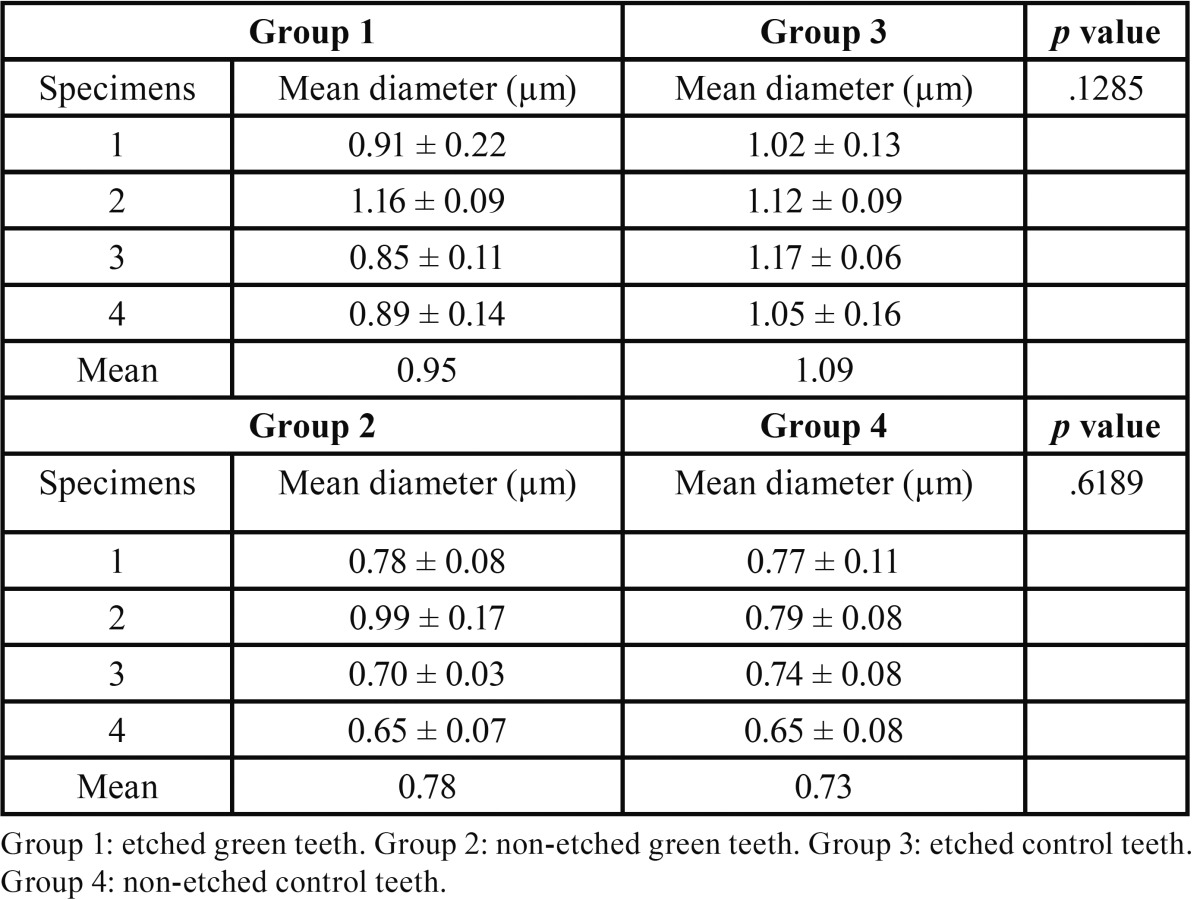
Mean diameter of dentin tubules.

**Table 4:**
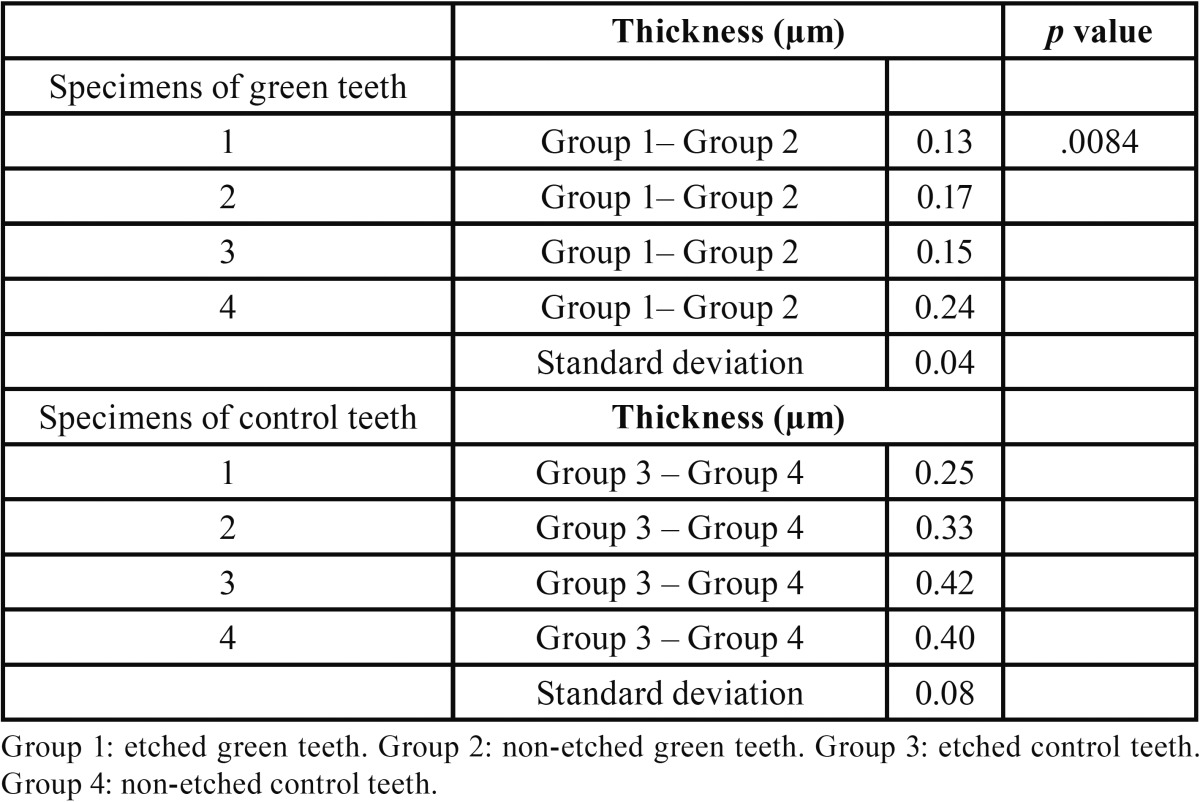
Thickness of peritubular dentin.

-Statistical analysis

The mean density of dentin tubules showed the greatest significant difference between Groups 1 and 3 (*p* < .01); however, the control group showed the highest density. The thickness of peritubular dentin in green teeth showed significant differences between the mean values for Groups 1 and 2 and Groups 3 and 4 (*p* < .01). Control teeth presented the highest mean thickness. The statistical analysis of the presence of hyperbilirubinemia on the mean diameter of dentin tubules compared between Groups 1 and 3, and on the mean diameter of dentin tubules compared between Groups 2 and 4 did not show differences (*p* > .05).

## Discussion

The current analyses demonstrated that hyperbilirubinemia led to a decrease in the density of the dentin tubules in the green teeth. Remarkably, the values found in the present study are similar to those previously described for normal human dentin both in permanent (20-23) and primary (24) anterior teeth; the number of tubules varied from 3.28 to 32.31 per square millimeter in such studies. Another study (25) compared tubule density between primary and permanent molars and suggested that tubule density was greater in primary molars (primary: 124.32/mm2 versus permanent: 45.97/mm2). Interestingly, this is the first study suggesting that high levels of bilirubinemia could yield a dentin with fewer tubules (26-28). The comprehension of the pathophysiological basis of the dentin changes secondary to hyperbilirubinemia is beyond the limits of the current study, but these data certainly open new avenues of potential research and therapy.

Conversely, hyperbilirubinemia did not alter the diameter of dentin tubules in green teeth. The studies examining permanent (20-23) and primary (24) teeth observed values ranging from 0.50 to 1.94 µm. It was showed that comparing the incisal, middle and cervical thirds, no statistically significant differences were observed in relation to diameter of coronal dentinal tubules of non-erupted deciduous incisors (24), based on results as showed previous, this study only evaluated dentin tubules of cervical third, which presented dentin-tubules measures similar to findings of Costa *et al.*, 2002 (24). Another study analyzed the diameter of dentin tubules in primary molars and found that tubules’ diameters ranged from 0.96 to 1.29 µm, suggesting that different anatomic groups of teeth may present similar values for the diameter of the tubules (25). The findings of lack of altered diameter of dentin tubules in this study represent an interesting perspective for treatment of patients who developed hyperbilirubinemia in childhood because it is known that the diameter of dentin tubules influence dental adhesion, since they are related to dentinal permeability, and the smaller diameter may account for the lower permeability, that is because the efficacy of most of the current adhesives depends on the infiltration of the resin into the dentin (22,24).

The control group of normal teeth specimens showed peritubular dentin with approximately twice the value of the thickness of peritubular dentine in green teeth (apparently, this is the first study to determine the thickness of peritubular dentine in primary teeth). This suggests that hyperbilirubinemia was able to reduce the thickness of peritubular dentin of green teeth probably affecting the physiology of odontoblasts because each odontoblast secrete additional noncollagenous components from the end of its cell process and this matrix mineralizes rapidly between the previously formed intertubular dentine and the odontoblast process, and constitutes the peritubular dentin, containing mostly apatite crystals with little organic matrix (29,30). In the context of the current study that was based on deciduous teeth samples, dentin changes related to hyperbilirubinemia certainly occurred during dentinogenesis, which was probably due to its negative impact on odontoblasts metabolism during dentin formation, a biological event previously suggested by several authors (2,3,5,6,26-29,31).

The observed lower densities of dentin tubules as well as the reduction of the thickness of peritubular dentine in green teeth may be a consequence of long-standing childhood hyperbilirubinemia, which may be able to alter the patterns of dentin mineralization during odontogenesis (3,4). The findings of this study highlighted the importance of tooth structure to dental treatment because bond strength used for evaluations of adhesive material performance are influenced by structural characteristics such as diameter and the number of dentinal tubules per mm2, as well as the relative amount of peritubular and intertubular dentin (25,30).

Based on the findings of the current study, it is possible that hyperbilirubinemia accounts for changes in dentin. It is also important to consider possible toxic effects of liver failure and PBC on teeth development and consequent dentin changes; however, none of the studied patients presented clinical evidence of retarded growth. The changes in the dentin of hyperbilirubinemia-related green teeth described herein seem to result from a compromised process of dentin mineralization. However, further studies are necessary to explain the involved mechanisms. Besides, a study based on teeth of affected patients after liver transplantation would be helpful to determine if normal bilirubin levels restore the function of the dentin-pulp complex and lead to a normal pattern of dentin tubules and peritubular dentin. This study supported the tested hypothesis that hyperbilirubinemia would be able to alter the morphology of dentin, and concluded that different liver diseases of childhood can alter the dentin morphology of green teeth, which is a secondary indicator for hyperbilirubinemia.
